# Development of a Candidate TMV Epitope Display Vaccine against SARS-CoV-2

**DOI:** 10.3390/vaccines12050448

**Published:** 2024-04-23

**Authors:** Kelvin Phiri, Larry Grill

**Affiliations:** Henry E. Riggs School of Applied Life Sciences, Keck Graduate Institute, Claremont, CA 91711, USA; larry_grill@kgi.edu

**Keywords:** SARS-CoV-2, epitope display vaccine, tobacco mosaic virus, *Nicotiana benthamiana*, spike protein

## Abstract

Essential in halting the COVID-19 pandemic caused by SARS-CoV-2, it is crucial to have stable, effective, and easy-to-manufacture vaccines. We developed a potential vaccine using a tobacco mosaic virus (TMV) epitope display model presenting peptides derived from the SARS-CoV-2 spike protein. The TMV-epitope fusions in laboratory tests demonstrated binding to the SARS-CoV-2 polyclonal antibodies. The fusion constructs maintained critical epitopes of the SARS-CoV-2 spike protein, and two in particular spanned regions of the receptor-binding domain that have mutated in the more recent SARS-CoV-2 variants. This would allow for the rapid modification of vaccines in response to changes in circulating variants. The TMV-peptide fusion constructs also remained stable for over 28 days when stored at temperatures between −20 and 37 °C, an ideal property when targeting developing countries. Immunogenicity studies conducted on BALB/c mice elicited robust antibody responses against SARS-CoV-2. A strong IFNγ response was also observed in immunized mice. Three of the six TMV-peptide fusion constructs produced virus-neutralizing titers, as measured with a pseudovirus neutralization assay. These TMV-peptide fusion constructs can be combined to make a multivalent vaccine that could be adapted to meet changing virus variants. These findings demonstrate the development of a stable COVID-19 vaccine candidate by combining SARS-CoV-2 spike protein-derived peptides presented on the surface of a TMV nanoparticle.

## 1. Introduction

The pandemic that has plagued the world in the past few years has been caused by one of nine identified coronaviruses (CoVs) that can infect humans [1]. Previously, severe acute respiratory syndrome CoV (SARS-CoV) and Middle Eastern respiratory syndrome CoV (MERS-CoV) were responsible for the heightened awareness of the potential for CoVs to cause a global pandemic [2]. The year 2019 saw the emergence of the Wuhan strain, now called SARS-CoV-2, which causes what is commonly known as coronavirus disease 2019 (COVID-19). Infection with SARS-CoV-2 typically leads to high mortality and morbidity [3]. Over 6 million people have died from SARS-CoV-2 infections since the pandemic started [4].

Although the mortality rate has steadily decreased since the height of the initial wave, the continued emergence of new variants hinders efforts to control the pandemic. There is still a pressing need to develop new, highly adaptable vaccines to meet the challenge posed by new viral strains as they develop. Currently, vaccines are only widely available to developed countries, leaving out an extensive reservoir of people in which the virus may spread and mutate, thus prolonging the eradication of the disease. Vaccines that are efficient, cost-effective, and stable in non-refrigerated conditions would benefit society. Different types of vaccines have been developed for SARS-CoV-2. This includes Adenoviral vector vaccines (Sputnik, Russia and Oxford-AstraZeneca vaccines), inactivated (Sinovac), nucleic acid-based vaccines (Moderna, Pfizer-BioNTech), and protein subunit vaccines (Novavax). SARS-CoV-2 has heralded unparalleled advancement in vaccine research and development due to the real-time sharing of research data and more available funding for research. All the vaccines and manufacturing technologies employed against COVID-19 have pros and cons. In addition to stimulating robust humoral and cellular immunity, vaccines need to cater to the speed of manufacturing scale-up, environmental stability, and delivery formulation.

**Figure 1:**
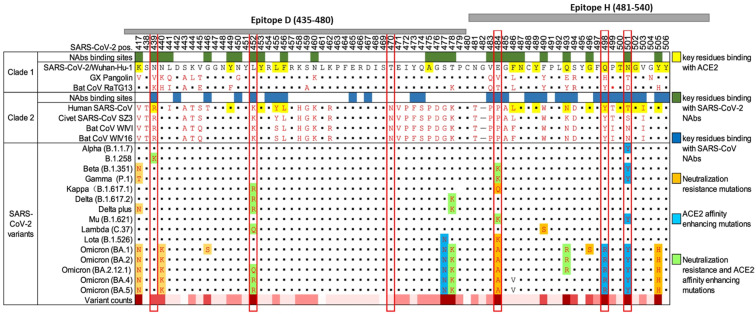
Landscape of amino acid mutations within the SARS-CoV-2 RBD. Alignment of the RBD residues SARS-CoV-2 variants is shown, highlighting key residues critical for binding by antibodies and ACE2. The grey bars at the top of the diagram represent two of the peptides used in this study in relation to the RBD and the mutations they cover. The chart was adapted from Yi (2022) [6].

Adaptive mutations in the viral genome can alter the virus’s pathogenic potential. This includes enhanced binding affinity to the cellular receptor ACE2 and the virus’s ability to evade the immune system, thus complicating the vaccine development process. SARS-CoV-2, like other RNA viruses, is prone to genetic evolution while adapting to new hosts with the development of mutations over time. Consequently, multiple variants with different characteristics compared to their ancestral strains may emerge. Throughout this pandemic, SARS-CoV-2 variants have been associated with enhanced transmissibility, reduced neutralization by antibodies, the ability to evade detection, or decreased effectiveness of therapeutics and vaccines. Five variants: Alpha (B.1.1.7); Beta (B.1.351); Gamma (P.1); Delta (B.1.617.2); and Omicron (B.1.1.529), have been identified, and they all have mutations in the receptor-binding domain of the spike protein [5] (Figure 1). These mutations have impacted vaccine development and efficacy. This rapid mutation rate needs to be considered when developing vaccines against SARS-CoV-2.

Nanoparticle (NP)-based vaccines can be delivered through various routes and elicit potent innate and adaptive immune responses in humans and animals. They are easily adapted to specific pathogens and can readily be changed in response to changing or emerging viral strains [7,8]. Tobacco mosaic virus (TMV) epitope display particles are one type of nanoparticle-based vaccine. This technology has previously proven clinically efficacious for non-Hodgkin’s lymphoma [9] and malaria [10,11]. The use of plants as a manufacturing platform for vaccines and therapeutics has been well documented, and many plant-manufactured products show great promise in preclinical testing [12,13,14]. A plant-made quadrivalent influenza virus vaccine completed Phase III clinical testing recently, and it compared favorably to a commercial quadrivalent vaccine [15,16]. Plant viruses are harmless to animals while being effective immunogens. This characteristic has been used to study the function and maturation of antigen-presenting cells (APCs). This characteristic of plant viruses, particularly TMV, is advantageous as TMV does have adjuvating effects when used as an epitope display particle in vaccines [17].

While one of the limitations of TMV genetic fusions is the size of the epitope that can be fused to the TMV coat protein [18], a helical linker allows larger peptides to be used [19]. The largest documented peptide successfully genetically fused to TMV was 120aa long [19]. TMV-based vaccines are potent activators of dendritic cells [20]. TMV epitope display technology is highly adaptable and can quickly be modified to meet the rapidly changing disease landscape [18,21,22]. Preclinical trials have shown that these vaccines are efficacious [16,23,24]. To compensate for the smaller peptides displayed, TMV epitope display vaccines lend themselves easily to mixing and matching epitopes into a multivalent vaccine. This makes these vaccines adaptable to rapidly mutating viruses, such as SARS-CoV-2.

Here, we described the development of a candidate TMV epitope display vaccine that displays epitopes from the SARS-CoV-2 spike glycoprotein (S). This protein binds to the cellular receptor ACE2 and facilitates viral fusion to the host cells [25]. We showed that the production of the vaccine is robust and that the candidate vaccine induced high neutralizing antibody titers in mice.

## 2. Methods

### 2.1. Peptide Design

Peptides were designed by considering the major immunodominant protein. For SARS-CoV-2, this is the S protein (Wuhan strain accession #NC_045512.2). B- and T-cell epitopes of the S protein have been mapped and published [26,27,28,29]. The epitopes used in this project utilized this knowledge when designing epitopes (Table 1). The simulation of the secondary and tertiary structure of the S-protein was conducted to predict possible antigenic portions of the S protein. The length of epitopes was limited to the length of the tertiary structure in which a known epitope resides. Also, an attempt to make each peptide multi-epitopic was made without making the new peptide too long to attach to the TMV-CP. Sixteen epitopes were designed (Table 1) for fusion to the TMV CP. S14P5 and S21P2 are published epitopes used in diagnostics and may have utility in vaccine development [30].

### 2.2. TMV Expression and Virus Purification

The TMV coat protein (CP) was genetically modified at the C-terminus through the addition of the SARS-CoV-2 epitopes we designed via a helical linker. The recombinant pJLTRBO plasmids were transformed into chemically competent *Agrobacterium tumefaciens* (GV3101) [31]. As Henderson et al. (2020) [32] indicated, the transformed Agrobacterium was vacuum infiltrated into *Nicotiana benthamiana* plants. After 14 days, the virus was extracted from leaf tissue as described in Chapman S.N. (1998) [33] with slight modifications to the extraction buffer composition (50 mM of sodium acetate, 0.1% sodium metabisulfite (*w*/*v*), 0.01% BME (*v*/*v*), and pH of 5.0). The viral particles were further purified via sucrose density gradient centrifugation as described in Bruckman and Steinmetz (2014) [34].

### 2.3. DLS Measurements on Viral Samples

Virus preparations were analyzed using DLS and Zeta potential techniques. Measurements were carried out using a Zetasizer Nano ZS device (Malvern Instruments Ltd., Worcestershire, UK) with a He-Ne laser (633 nm, 10 mW) as a light source. Polystyrene cells with 10 mm optical paths were used for the DLS experiments, where sample volumes were 1 mL and the concentration of virus preparation was in the range of 0.15–0.3 mg/mL [35].

### 2.4. SDS-PAGE, Western Blot, and ELISA

For SDS–PAGE, purified TMV virus extracts were prepared 1:1 in Laemmli sample buffer (BioRad, Hercules, CA, USA #1610737) with beta-mercaptoethanol, boiled, and run on 4–20% Mini-Protean TGX gels (BioRad #4561096) in 1× Tris/Glycine/SDS buffer and stained with BioSafe Coomassie G-250 stain (BioRad #1610787) according to the manufacturer’s instructions. Western blot analysis was performed as in McComb et al. (2015) [36], with the following modifications. Primary antibodies were diluted in blocking buffer at 1:3000 (Rabbit anti-SARS-CoV-2 spike polyclonal, MyBioSource, San Diego, CA, USA #MBS434243) or 1:500 (Rabbit anti-TMV polyclonal, Agdia, Elkhart, IN, USA #57400). Secondary antibodies were diluted at 1:3000 (Goat anti-Rabbit HRP, BioRad #1706515). Opti-4CN (BioRad #1708235) reagent was added for 5 min for detection. Images were acquired using a 5-megapixel camera. For Indirect ELISA analysis, 96-well plates were coated with 1 µg of the SARS-CoV-2 spike protein (MyBioSource #MBS1560493) or 1 µg of the purified TMV virus in 100 mM of Bicarbonate/Carbonate buffer (0.03 M of Na_2_CO_3_/0.07 M of NaHCO_3_, and a pH of 9.6) overnight at 4 °C. The primary antibodies were diluted in 5% non-fat milk in TBST and incubated at 4 °C overnight. Secondary antibody dilutions were incubated at room temperature for 2 h. O-phenylenediamine dihydrochloride (OPD) substrate (Thermo Scientific™, Waltham, MA, USA #34006) was used according to the manufacturer’s instruction, developed for 30 min, and read at 450 nm. For serum antibody analyses, mouse serum samples were diluted 3-fold beginning at 1:100 in 5% non-fat milk in phosphate-based saline. Goat anti-mouse IgG HRP (BioRad #172-1011) was used at 1:3000 for secondary antibody detection. Antibody midpoint titers were determined in a GraphPad prism by plotting the absorbances from the ELISA dilution series and fitting a dose–response curve using the four-parameter logistic (4PL). The IC50 values obtained are the midpoint titers. The data were analyzed using Microsoft Excel, Version 2403 Build 16.0.17425.20176. Graphs were created and statistical analyses between groups were conducted using GraphPad Prism 9.3.1.

### 2.5. Antigenicity Study

**Figure 2:**
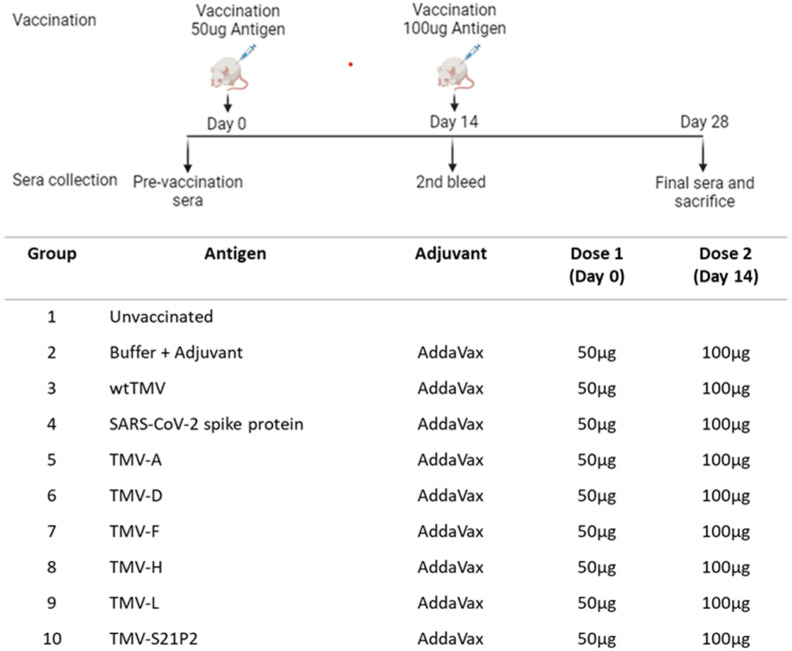
Schematic diagram of injection and bleed schedule. The immunization regime and dosing schedule in BALB/c mice vaccinated with 50 μg of recombinant TMV formulated with AddaVax™ is depicted. All vaccine formulations were administered as a 100 μL dose subcutaneously (s.c.). The preimmune bleed was conducted a day before the 1st dose on day 0. The second bleed was conducted on day 14, prior to administering the booster. Day 28 was the final bleed. The vaccination groups are shown in the table.

All animal experiments followed IACUC standards at Pocono rabbit farms and laboratory. Seven-week-old female BALB/c mice received two subcutaneous injections two weeks apart on days 0 (50 µg) and 14 (100 µg) of SARS-CoV-2 S (MyBioSource # MBS1560493), TMV-(A, D, F, H, L, S21P2), wtTMV, and buffer (0.01 M phosphate buffer, pH 7.2) + adjuvant. All samples were mixed 1:1 *v*/*v* with AddaVax™ (InvivoGen, San Diego, CA, USA #vac-adx-10) to make an injectable volume of 100 µL. Serum was harvested from anesthetized mice (five mice per group) in the bleed groups by taking blood samples from the retro-orbital sinus one day before each vaccination (days 0, 14, and 28) (Figure 2)

### 2.6. IFN-γ Detection with ELISA

To investigate the level of IFN-γ production in mice, the IFN-γ ELISA kit from Abcam, Cambridge, UK (ab100689 IFNγ Mouse ELISA Kit) was used following the manufacturer’s instructions. In short, 100 μL of each recombinant mouse IFN-γ standard and sera from immunized mice were added into wells and incubated overnight at 4 °C with gentle shaking. A total of 100 μL of biotinylated IFN-γ detection antibody was added to each well after four washes and incubated for 1 h at room temperature. In total, 100 μL of HRP-Streptavidin solution was then added and incubated for 45 min at room temperature, followed by 100 μL of TMB One-Step Substrate Reagent to each well and incubated for 30 min at room temperature in the dark with gentle shaking. After incubation, 50 μL of stop solution was added to each well, and the results were read at 450 nm. A standard curve was generated and used to calculate the concentration of the test sera. A GraphPad prism was used to plot the graph of the IFN-γ concentration, and one-way ANOVA was used to analyze the difference between wtTMV and TMV(-A, -D, -F, -H, -L, and -S21P2).

### 2.7. Neutralization

Neutralization titers were determined using the COVID-19 pseudovirus Neutralizing Antibody Assay (Luciferase) (Abnova, Taipei City, Taipei, Taiwan #KA6152) following the manufacturer’s instructions. 1 × 10^5^ cells/well HEK293T-ACE2 cells in DMEM (10% FBS) were seeded in 24-well plates 5 h before beginning the neutralization assay. In a separate microcentrifuge tube, 50 µL of the diluted sample (either mouse serum or mAb) and 20 µL of pseudovirus expressing luciferase were mixed and incubated at room temperature for 30 min. The mixture was added to wells containing HEK293-ACE2 cells, and the plates were incubated at 37 °C for 48 h. Media from each well were removed, and cells were washed with 200 µL of PBS. A total of 150 µL of Luciferase Cell Culture Lysis Reagent was used to harvest cells from plates, and 10 µL of this lysate was mixed with 50 µL of Luciferase Assay Reagent in 96-well plates. A luminescence microplate reader was used to detect the luciferase expression. IC50 was calculated using the method outlined in Ferrara and Temperton (2018) [37]

### 2.8. Thermal Stability

The thermal stability of the three recombinant TMV constructs that showed neutralization was tested by incubating 100 µg (100 µL) in 0.01 M of phosphate buffer (pH of 7.2) samples at −20 °C, 4 °C, 25 °C, and 37 °C for 28 days. On the 28th day, the samples were run on an SDS-PAGE gel and stained with Coomassie blue (BioRad #1610787), and the resultant bands were analyzed for protein degradation.

## 3. Results

### 3.1. TMV Expression and Virus Purification

**Figure 3:**
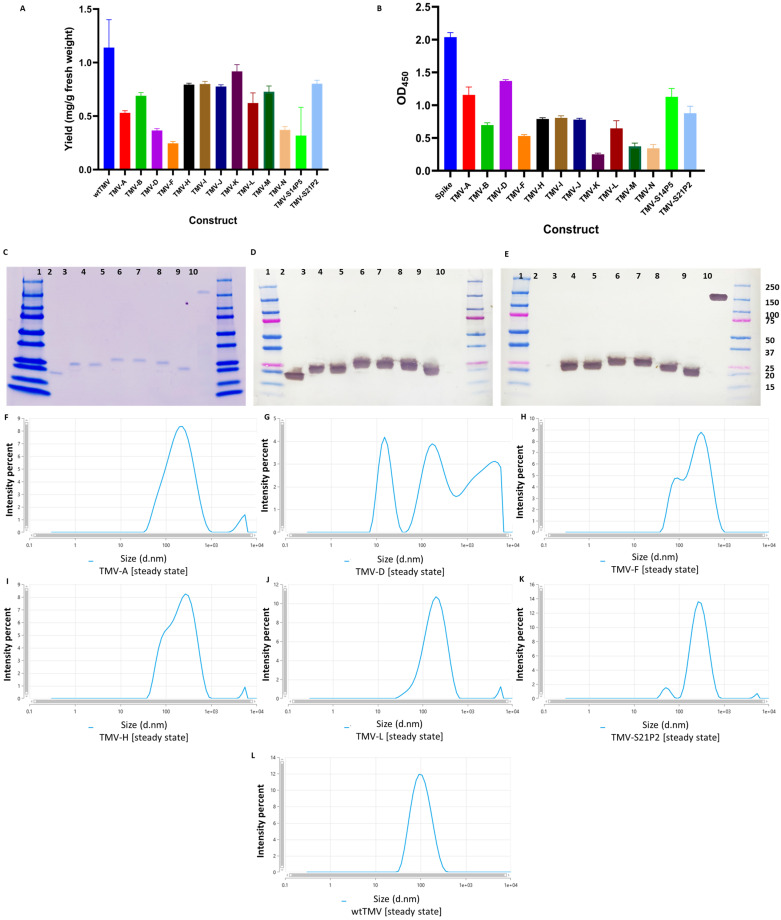
Analysis of purified TMV-epitope constructs. (**A**) Comparison of the average virus yield per construct. Purified recombinant TMV was quantified using absorbance at 280 nm, and the yield was calculated relative to fresh plant matter. (**B**) ELISA results showing the antigenicity of the TMV-epitope constructs. A 96-well plate was coated with purified TMV epitope. Rabbit anti-SARS-CoV-2 spike polyclonal antibody was used as the primary antibody, and the ELISA was developed using OPD. The images show an analysis of six of the TMV-epitope constructs using SDS-PAGE (**C**). The Western blots were carried out using either anti-TMV (**D**) or anti-SARS-CoV-2 S polyclonal antibody (**E**). The blots were loaded as follows: Lane 1: prestained molecular weight marker (BioRad), Lane 2: wtTMV, Lane 3: TMV-A, Lane 4: TMV-D, Lane 5: TMV-F, Lane 6: TMV-H, Lane 7: TMV-L, Lane 8: TMV-S21P2, Lane 9: SARS-CoV-2 spike, and Lane 10: prestained molecular weight marker (Biorad). (**F**–**L**) Size distributions that reflect their aggregation state rather than the absolute hydrodynamic radius of the TMV constructs.

Classic symptoms of TMV infection appeared within 14 days post-infiltration (dpi) for all the constructs. Purified virus extracts were analyzed using SDS–PAGE, revealing the expected size for all the constructs in Table 1. TMV-C, -E, and -G were toxic to *N. benthamiana* plants and killed the plants within 2–3 dpi; hence, no virus could be purified. Yields of wtTMV averaged above 1 mg virus/g of fresh leaf weight. The average yield was calculated from five different extractions from 10 plants. The yields from the TMV-epitope fusions varied significantly. TMV-F had the lowest yield (0.3 mg/g), while TMV-K L14 had the highest (0.8 mg/g). There was minimal variation in the yield between extractions (Figure 3A). Only six of the constructs that could be extracted were selected for further analysis. The selection was based on the binding affinity to SARS-CoV-2 spike protein polyclonal antibody, yield, and permission (S14P5). TMV-B and TMV-L scored equally on this criterion but the proximity of the L-peptide to the SARS-CoV-2 spike protein furine cleavage site tilted the decision in its favor [38]. Western blot with anti-TMV coat protein antibody confirmed the identity of extracted coat protein monomers and their respective sizes (Figure 3). All six TMV-epitope constructs selected for the animal study showed cross-reactivity with polyclonal sera raised in rabbits against TMV (Figure 3D) and SARS-CoV-2 spike protein (Figure 3E). SDS-PAGE and Western blot analysis of the other constructs are shown in the Supplementary Materials. Not surprisingly, neither the wtTMV coat protein nor the SARS-CoV-2 spike protein showed cross-reactivity with anti-S protein or anti-TMV antibodies, respectively (Figure 3D,E).

An ELISA was employed to detect the SARS-CoV-2 peptides on the TMV CP surface. To allow the detection of the fused peptides, each TMV-epitope fusion was used to coat ELISA plates, which were then probed with anti-SARS-CoV-2 spike protein polyclonal antibodies. The binding affinity of the epitopes was analyzed by observing the absorbance of the different constructs in the ELISA. The binding affinity is directly proportional to the absorbance [39]. All the constructs showed varying levels of binding affinity to the anti-SARS-CoV-2 spike protein polyclonal antibodies. TMV-A, TMV-D, and TMV-S14P5 showed the highest binding affinity, while TMV-K, TMV-M, and TMV-N had the lowest (Figure 3B)

The aggregation observed for the TMV(-A, -D, -F, -H, -L, and -S21P2) (Figure 3F–K) samples in the DLS data suggested that these samples had a distribution of larger particles than the wtTMV (Figure 3L). The analysis of the hydrodynamic diameter of particles in solution using DLS showed that the unmodified TMV preparation had particles with an average size of about 100 nm, which in general coincides with the DLS data obtained for the other helical plant viruses [35]. In contrast, the TMV constructs appeared as complexes with an average size of about 200 nm, which indicates that this virus is in an aggregated state in solution. Despite the fact that DLS is used primarily in the analysis of globular particles, it is also possible to use in the study of rod-shape viruses, especially where the aggregation states of the viruses are being considered rather than their absolute hydrodynamic radius.

### 3.2. Antigenicity Study

#### 3.2.1. Vaccine Immunogenicity

**Figure 4:**
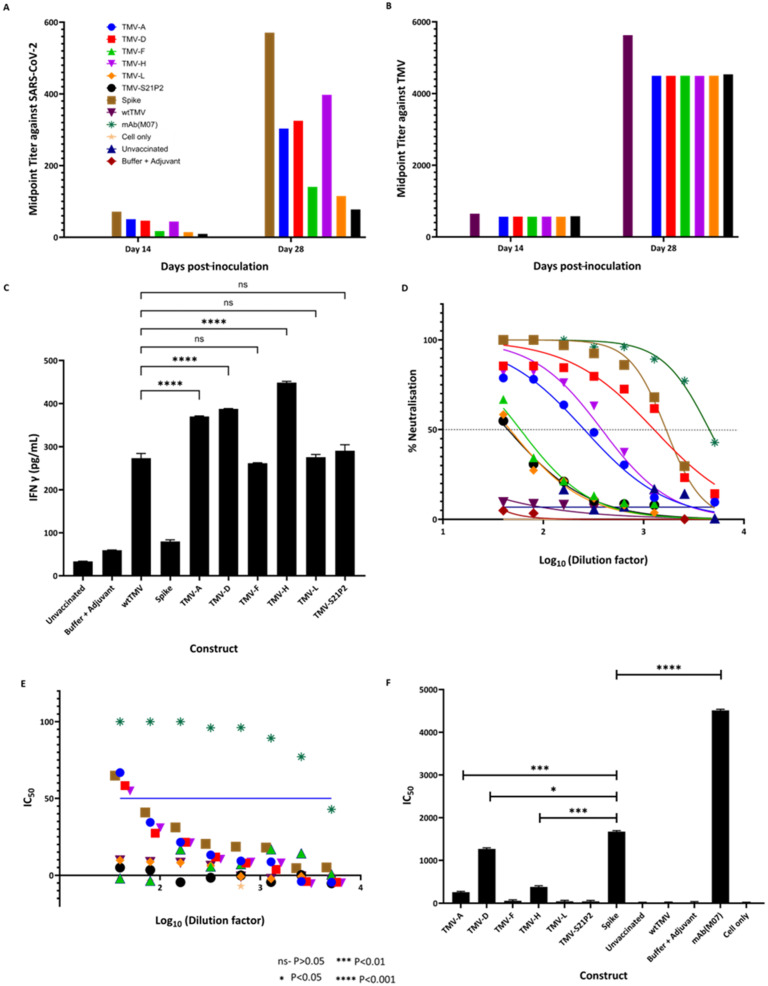
Antibody quantification using midpoint titers against (**A**) SARS-CoV-2 spike protein and (**B**) against TMV using sera from mice immunized with wtTMV, TMV-A, TMV-D, TMV-F, TMV-H, TMV-L, and TMV-S21P2 at 14 and 28 dpi. SARS-CoV-2 spike protein/wtTMV-coated ELISA plates were incubated with serum samples taken at various points during the vaccination schedule (preimmune—day 0, two weeks following vaccination—day 14, two weeks following the first bleed—day 28). Midpoint titers were calculated and plotted using GraphPad prism, with (**C**) showing the estimated IFN-γ concentrations (pg/mL) in mice 28 days post-inoculation. Sera from mice were used to coat wells in an ELISA plate and probed with an anti-IFN-γ antibody. The concentration of IFN-γ in the mice sera was extrapolated from a standard curve generated from known IFN-γ concentrations. (**D**) The percentage neutralization of sera taken 28 dpi. Neutralization was carried out using a pseudovirus assay (Abnova). The IC_50_ (**E**) was calculated from the neutralization assay. The blue line shows the concentration at which half neutralization occurs, and the IC_50_ dilution titers are shown in (**F**). All the data analyses were performed using GraphPad Prism. The key for the color coding in the graphs is shown in Figure (**A**). In the colored graphs, the color code is as follows: Blue—TMV-A; Red—TMV-D; Green—TMV-F; Pink—TMV-H; Orange—TMV-L; Black—TMV-S21P2; Tan—Spike; Maroon—wtTMV; Dark green—mAb(M07), Yellow—Cell Only; Navy Blue—Unvaccinated; Dark red—Buffer + Adjuvant.

The ability of our candidate vaccines to promote an immune response targeting SARS-CoV-2 and TMV was assessed in female BALB/c mice. Mice were immunized subcutaneously (s.c.) with one of the candidate vaccine constructs on days 0 (50 µg) and 14 (100 µg). The vaccine was formulated with AddaVax at a 1:1 *v*/*v* ratio in a final dose volume of 100 µL. Blood was drawn for antibody analysis on days 0, 14, and 28. The serum was tested for total IgG reactivity against SARS-CoV-2 spike protein and wtTMV via ELISA, utilizing histidine-tagged S1 (S1-6His) from SARS-CoV-2 (Figure 4A) or wtTMV (Figure 4B) as the capture antigen. The IgG titers are represented as midpoint titers.

All mice vaccinated with the TMV-epitope fusions showed cross-reactive IgG antibodies to SARS-CoV-2 spike protein by day 28 (Figure 4A). Serum from mice immunized with wtTMV showed no reactivity to SARS-CoV-2 spike protein (Figure 4A), while serum from mice vaccinated with SARS-CoV-2 spike protein showed no reactivity to wtTMV (Figure 4B). SARS-CoV-2 IgGs were present in all the sera from mice immunized with the TMV-epitope fusions on day 14 post-vaccination, and the titers increased by day 28 (Figure 4A), except for mice vaccinated with TMV-S21P2, which showed a very low titer on both day 14 and day 28 (Figure 4A). TMV-specific midpoint titers in vaccinated sera were higher than the titers observed for SARS-CoV-2-specific antibodies. The TMV-specific antibody midpoint titers were also uniform for each sample day (Figure 4B).

#### 3.2.2. IFN-γ Detection

The vaccination of mice with the TMV-epitope fusions resulted in an inflammatory response measured in the amount of IFN-γ 28 dpi (Figure 4C). ELISA determined IFN-γ concentrations and compared concentrations to those of known standards. Although AddaVax was used as an adjuvant, the constructs elicited a much higher IFN-γ response. wtTMV also had significantly lower IFN-γ levels than the fusion constructs. TMV (-A, -D, and -H) had the highest levels of IFN-γ, while the other three had levels similar to those of wtTMV.

#### 3.2.3. Neutralization

IgG titers are an essential measure of vaccine performance; however, that alone does not directly translate to protection. More important is the ability of a vaccine to elicit a neutralizing immune response that protects the host by preventing viral binding or entry into host cells. We performed a neutralization assay using a COVID-19 Pseudovirus Neutralizing Antibody Luciferase Assay (Abnova #KA6152). This assay provides a highly sensitive and specific method of quantifying COVID-19-neutralizing antibodies. It consists of neutralizing the antibody blockade of lentivirus pseudotyped with SARS-CoV-2 spike protein (Wuhan strain), with the luciferase (Luc) reporter gene interacting with ACE2-expressing HEK293T cells (HEK293T-ACE2). The Luc expression inside the HEK293T-ACE2 signifies viral entry and no neutralizing antibody activity. In contrast, the absence of Luc expression signifies the presence of neutralizing antibody. The Luc reporter system enables the high-sensitivity measurement of antibody neutralization. Here, we showed that sera from mice immunized with TMV-A (IC_50_—256.2), TMV-D (IC_50_—1270.0), and TMV-H (IC_50_—383.0) elicited a high titer of neutralizing antibodies (Figure 4E).

Figure 4D shows the percentage neutralizing antibody titer at which IC50 is obtained. Only sera from mice immunized with TMV-A, TMV-D, and TMV-H showed greater than 50% neutralization at higher dilutions. While sera from mice immunized with TMV-F, TMV-L, and TMV-S21P2 only showed greater than 50% neutralization in the first dilution. Sera from mice vaccinated with the spike protein had a higher neutralizing antibody titer than the TMV-peptide fusions. IC_50_ is a crucial parameter used to evaluate the efficacy of antibodies in preventing the replication or infectivity of a specific pathogen. It represents the concentration of antibody needed to inhibit the pathogen’s activity by 50%. Here, Figure 4F shows the IC50 for each of the constructs and controls. The constructs TMV-A (IC_50_—256.2), TMV-D (IC_50_—1270.0), and TMV-H (IC_50_—383.0) were neutralizing.

#### 3.2.4. Thermal Stability

**Figure 5:**
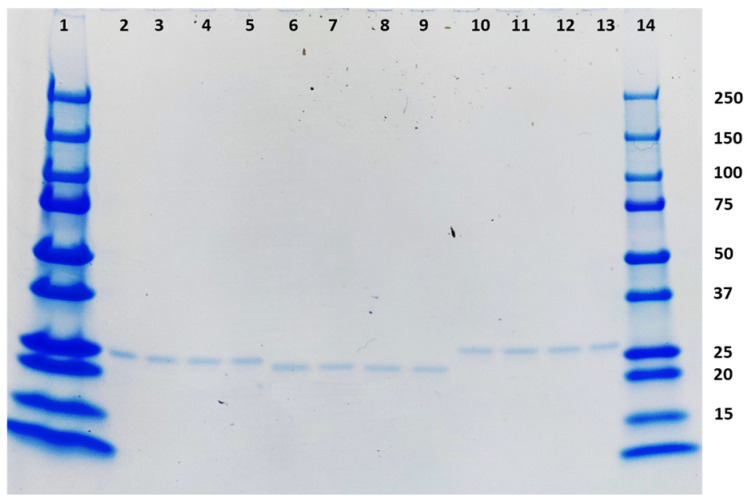
Coomassie-stained SDS-PAGE showing the stability of the three TMV-epitope fusions that elicited neutralizing antibodies in mice after storage at −20 °C, 4 °C, 25 °C, and 37 °C for 28 days. The loading order is as follows: Lane 1: Prestained molecular weight marker (BioRad), Lanes 2–5: TMV-A (−20 °C, 4 °C, 25 °C, and 37 °C), Lanes 6–9 TMV-D (−20 °C, 4 °C, 25 °C, and 37 °C), Lanes 10–13: TMV-H (−20 °C, 4 °C, 25 °C, and 37 °C), Lane 14: prestained molecular weight marker (BioRad).

The three TMV-epitope fusions that elicited neutralizing antibodies in mice were stored at various temperatures. After 28 days of storage, all the samples did not show any degradation when analyzed in an SDS-PAGE using Coomassie staining (Figure 5).

## 4. Discussion

In the COVID-19 vaccine response, developing countries were among the last to receive vaccines, relying on other countries for vaccine development and high-technology production. Utilizing greenhouse-grown plants to produce vaccines provides several benefits that developing countries can use. Production can be rapid, with plants yielding large amounts of the vaccine in approximately a week. The cost of building large plant growth rooms is substantially lower than the expensive production facilities for the current FDA-approved COVID-19 vaccines. In addition, the cost of extracting the vaccines from the plants is also not overly expensive. The development and production of new vaccines for outbreaks and pandemics are within the capabilities of many developing countries. The thermal stability of the plant-produced vaccines will also be advantageous for new vaccines. Several studies have shown that TMV epitope display particles are stable for long periods at 4 °C [21,32,36]. This is one of the limiting factors hindering the widespread use of the highly successful mRNA vaccines currently on the market [40]. TMV epitope display vaccine technology is a step toward providing thermostable vaccines that cut down on the logistical problems posed by the need for cold-chain management.

Here, we report a platform capable of producing transiently expressed recombinant antigens as TMV epitope display particles in *N. benthamiana*. We also show the simple purification of the recombinant TMV particles using PEG precipitation and sucrose gradient density centrifugation, to achieve a very high level of purity. The yields obtained are relatively high and consistent over multiple extractions. This lends itself well to ease of production and scale-up. The length of peptide used in the TMV-CP fusions is constrained by the need to maintain the integrity of the TMV-CP. TMV-CP fusions have been shown to be affected by peptide length, charge, and sometimes isoelectric point. These factors interfere with the expression, folding, and assembly of the TMV-CP [41]. DLS data showed that the particle sizes of the constructs increased compared to wtTMV. This is indicative of the increased molecular mass and physical size of the recombinant TMV generated by the epitope fusion [35]. To alleviate this problem, we included a helical linker that has been used to successfully express a 120-amino acid peptide on the surface of TMV [19]. Through the genetic fusion of the SARS-CoV-2 antigens to the TMV particle, we may have augmented the stimulation of the immune system compared to what is achievable with peptides (SARS-CoV-2 antigens) alone. This could be proved empirically by immunizing mice under the same conditions as the samples used in this study. However, TMV epitope display technology has advantages such as enhanced immunogenicity, longer immunological memory, efficient antigen presentation, stability, scalability, and customization [42,43]. Using short peptides allows for adapting a multivalent formulation in response to mutations to the SARS-CoV-2 spike protein. An example is the emergence of the Brazil, South Africa, Delta, and Omicron variants that all have mutations in the spike protein RBD, an area covered by peptides D and H used in this study. Changing the sequence of just those two peptides would potentially elicit a variant-specific, protective immune response. The utility of immunity against circulating variants of SARS-CoV-2, induced by the TMV-D and TMV-H constructs, can be investigated using the growing repertoire of SARS-CoV-2 variant-specific pseudoviruses [44,45].

TMV epitope-display vaccines have been shown to be stable at various temperatures [32,46]. Here, we showed that the recombinant TMV particles we made were stable for four weeks at 0 °C, 4 °C, 25 °C, and 37 °C. One of the biggest challenges that hamstring vaccination campaigns in developing countries is maintaining the cold chain in remote areas. The areas that remain largely unvaccinated are potential reservoirs of pathogens that are the source of continued outbreaks. A vaccine that would remain viable and efficient over a long period would help resolve this issue. With COVID-19, most vaccines require stringent cold temperatures of at least 4 °C while the mRNA vaccines (Moderna and Pfizer) have even more rigid temperature requirements to maintain viability [47,48].

We selected the S protein as it is the dominant target for neutralizing response in COVID-19 infection [28,49]. We used short peptides because the full-length S protein of other coronaviruses has been implicated in the enhancement of viral infection or pulmonary toxicity in other studies [50].

While all the candidate vaccines injected into mice elicited a strong IgG response against SARS-CoV-2 spike glycoprotein, not all candidate vaccines elicited a neutralizing antibody response. Three of the eight candidates in the mouse trials elicited high neutralizing antibody titers. As expected, the positive control showed the highest percentage neutralization. Although high, sera from mice immunized with the SARS-CoV-2 spike protein, the percentage of neutralization and IC_50_ was lower than that of the kit’s control. This could be because of the nature of the antibody, the lack of glycosylation in the spike protein used to immunize mice, or the general specificity of the positive control that renders its efficacy much higher than test sera.

Similarly, the sera from mice immunized with TMV-A, TMV-D, and TMV-H only elicited a fraction of neutralizing antibodies compared to the control; however, the constructs did elicit a neutralization of between 70 and 100% at lower dilutions. Although in vitro assays to detect neutralizing antibodies cannot completely predict the in vivo protection afforded by a vaccine, such assays are helpful for screening vaccine candidates. This suggests that a multivalent formulation containing these three candidates might elicit a strong, protective immune reaction [51]. As with previous TMV epitope display vaccines and TMV-based VLPs, the presence of the TMV particle has been shown to elicit a potent cellular and humoral immune response [52]. We suggest that this formulation would perform similarly, which is important for SARS-CoV-2 as both arms of the immune system are essential for viral clearance and protection. As shown in other TMV epitope display vaccines, there is a strong antibody response generated against TMV. In this study, the TMV-specific antibodies elicited by the different constructs were relatively even. This is because the backbone of the constructs is TMV CP and at equal concentrations, the level of TMV-specific antibody produced is similar [32,36]

Testing for IFN-γ levels in serum at the two-week post-immunization point is because IFN-γ is a central cytokine that orchestrates immune responses, particularly in cellular immunity and Th1-type immune reactions, by activating macrophages, enhancing antigen presentation, and regulating T-cell differentiation. Monitoring IFN-γ levels two weeks post-vaccination provides valuable insights into the effectiveness of the immune response triggered by the immunization, especially during the critical period when the adaptive immune response, including the development of memory T cells and antibody production, begins to peak. Assessing IFN-γ levels at this stage helps evaluate the early activation and functionality of the immune system in response to the vaccine. Studies have highlighted the importance of IFN-γ measurement in understanding vaccine efficacy, immune system activation, and predicting immune responsiveness [53,54]. In particular, for SARS-CoV-2, vaccine-induced protection requires an IFN-γ-driven cellular immune response [55,56]. In this study, high levels of IFN-γ were observed in mice immunized with TMV (-A, -D, and -H), suggesting that these constructs may elicit a robust T-cell response that may activate the cellular arm of the immune system. While the IFN-γ response studied here is not antigen-specific, the difference between the IFN-γ levels observed in the wtTMV + adjuvant group indicates that the increased levels observed are due to the SARS-CoV-2 epitopes, A, D, and H in particular, fused to the TMV-CP in the coat protein fusion vaccines. An investigation of antigen-specific IFN-γ would further support this hypothesis.

Since the start of the COVID-19 pandemic, vaccine development has accelerated at a very high rate. Vaccines using different platforms have emerged, and even more technologies are being developed. These technologies include vaccines based on inactivated viruses, nucleic acid (mRNA and DNA), recombinant spike proteins (including those presented as VLPs), as well as replication-competent or -incompetent virus vectors [47,48,57]. Recently, the utilization of the 1018 CpG or AS02 adjuvant with a plant-based NP vaccine elicited high neutralizing antibody titers in humans [58]. As such, we used AddaVax, an adjuvant in the same class of adjuvants (squalene-based, oil-in-water nano-emulsion). The successful production of neutralizing antibodies provided here shows the utility of plant-based vaccine technology. When coupled with positive safety data, it suggests that NP vaccines could play a role in combating SARS-CoV-2 by eliciting a robust immune response.

## Figures and Tables

**Table 1:** Epitopes used in the study.

Name	Position	Epitope Sequence	Mass (kDa)	
			Epitope	Epitope + TMV CP
A	300–330	KCTLKSFTVEKGIYQTSNFRVQPTESIVRFP	6.29	24.19
B	365–395	YSVLYNSASFSTFKCYGVSPTKLNDLCFTNV	4.16	22.06
C	420–440	DYNYKLPDDFTGCVIAWNSNN	3.05	20.95
D	435–480	AWNSNNLDSKVGGNYNYLYRLFRKSNLKPFERDISTEIYQAGSTPC	6.02	23.92
E	420–500	DYNYKLPDDFTGCVIAWNSNNLDSKVGGNYNYLYRLFRKSNLKPFERDISTEIYQAGSTPCNGVEGFNCYFPLQSYGFQPT	10.01	27.91
F	440–500	NLDSKVGGNYNYLYRLFRKSNLKPFERDISTEIYQAGSTPCNGVEGFNCYFPLQSYGFQPT	7.69	25.59
G	420–540	DYNYKLPDDFTGCVIAWNSNNLDSKVGGNYNYLYRLFRKSNLKPFERDISTEIYQAGSTPCNGVEGFNCYFPLQSYGFQPTNGVGYQPYRVVVLSFELLHAPATVCGPKKSTNLVKNKCVN	14.33	32.23
H	481–540	NGVEGFNCYFPLQSYGFQPTNGVGYQPYRVVVLSFELLHAPATVCGPKKSTNLVKNKCVN	7.28	25.18
I	475–500	AGSTPCNGVEGFNCYFPLQSYGFQPT	3.47	21.37
J	520–540	APATVCGPKKSTNLVKNKCVN	2.86	20.76
K	660–680	YECDIPIGAGICASYQTQTNS	2.92	20.82
L	660–710	YECDIPIGAGICASYQTQTNSPRRARSVASQSIIAYTMSLGAENSVAYSNN	6.12	24.02
M	990–1035	EVQIDRLITGRLQSLQTYVTQQLIRAAEIRASANLAATKMSECVLG	5.75	23.65
N	931–970	IGKIQDSLSSTASALGKLQDVVNQNAQALNTLVKQLSSNF	4.18	22.08
S21P2	709–727	PSKPSKRSFIEDLLFNKV	2.8	20.7
S14P5	552–570	TESNKKFLPFQQFGRDIA	2.8	20.72

TMV coat protein (17.9 kDa).

## Data Availability

Data are contained within article.

## Supplementary Materials

The following supporting information can be downloaded at https://www.mdpi.com/article/10.3390/vaccines12050448/s1.

## Abbreviations

SARS-CoV-2: severe acute respiratory syndrome coronavirus 2; COVID: coronaviral disease; TMV: tobacco mosaic virus; NAb: neutralizing antibody; S: spike; RBD: receptor-binding domain; IC50: half-maximal inhibitory concentration.
