# Supplementary material for: Development of a Candidate TMV Epitope Display Vaccine against SARS-CoV-2
Source: Vaccines (Basel). 2024 Apr 23;12(5):448. doi: 10.3390/vaccines12050448 (PMC11125883; doi:10.3390/vaccines12050448)
Supplement: Supplementary file 1 [file vaccines-12-00448-s001.zip › vaccines-2949740-supplementary.pdf]

Supplementary Material.

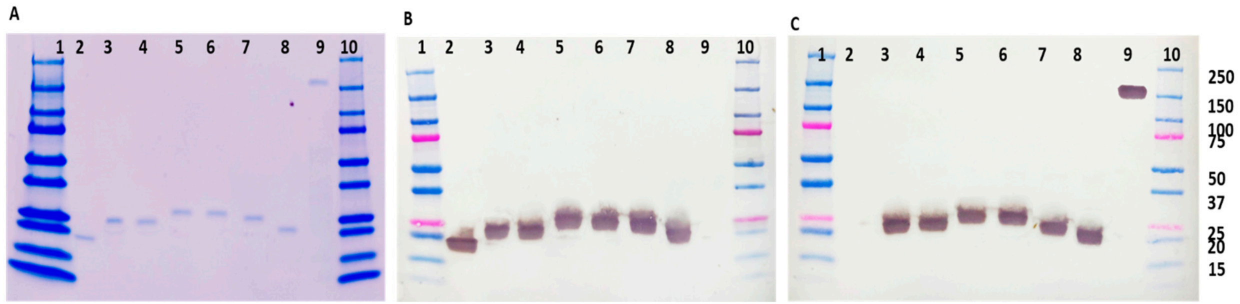

Figure S1. The images show analysis of the TMV-epitope constructs using SDS-PAGE (A); western blots were carried out using either anti-TMV (B); or anti-SARS-CoV2 S (C) polyclonal antibody. The blots were loaded as follows: Lane 1: Prestained molecular weight marker (BioRad). Lane 2: TMV-A, Lane 3 TMV-D, Lane 4: TMV-F, Lane 5: TMV-H, Lane 6: TMV-H, Lane 7: TMV-L, Lane 8: TMV-S21P2, Lane 9: SARS-CoV2 spike protein, Lane 10:. Ladder

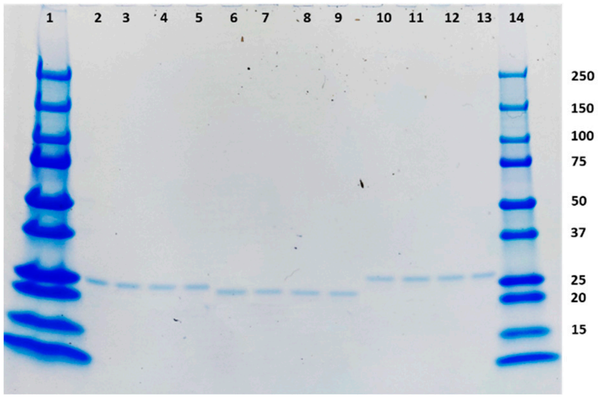

Figure S2. Coomassie-stained SDS-PAGE showing the stability of the three TMV-epitope fusions that elicited neutralizing antibodies in mice after storage at -20°C, 4°C, 25°C and 37°C for 28 days. The loading order is as follows: Lane 1: Prestained molecular weight marker (BioRad). Lane 2-5: TMV-A (-20°C, 4°C, 25°C, 37°C), Lane 6-9 TMV-D(-20°C, 4°C, 25°C, 37°C) , Lane 10-13: TMV-H(-20°C, 4°C, 25°C, 37°C Lane 14: Prestained molecular weight marker (BioRad)

Table S2. Protein and nucleotide sequences of epitopes used in this study

Name	Position	Epitope Sequence
A	300-330	KCTLKSFTVEKGIYQTSNFRVQPTESIVRFP
		aagtgtacgttgaaatccttcactgtagaaaaaggaatctatcaaacttctaactttagagtccaaccaacagaatctattgtta<br>gatttccttag
B	365-395	YSVLYNSASFSTFKCYGVSPTKLNDLCFTNV
		tattctgtcctatataattccgcatcattttccacttttaagtgttatggagtgtctcctactaaattaaatgatctctgctttactaa<br>tgtctag
C	420-440	DYNYKLPDDFTGCVIAWNSNN
		gattataattataaattaccagatgattttacaggctgcgttatagcttggaattctaacaattag
D	435-480	AWNSNNLDSKVGGNYNYLYRLFRKSNLKPFERDISTEIYQAGSTPC
		gcttggaattctaacaatcttgattctaaggttggtggtaattataattacctgtatagattgtttaggaagtctaatctcaaacct<br>tttgagagagatatttcaactgaaatctatcaggccggtagcacaccttgttag
E	420-500	DYNYKLPDDFTGCVIAWNSNNLDSKVGGNYNYLYRLFRKSNLKPFERDISTEIYQAGSTPCNGVEGF<br>NCYFPLQSYGFQPT
		gattataattataaattaccagatgattttacaggctgcgttatagcttggaattctaacaatcttgattctaaggttggtggtaat<br>tataattacctgtatagattgtttaggaagtctaatctcaaaccttttgagagagatatttcaactgaaatctatcaggccggtag<br>cacaccttgtaatggtgttgaaggttttaattgttactttcctttacaatcatatggtttccaacccacttag
F	440-500	NLDSKVGGNYNYLYRLFRKSNLKPFERDISTEIYQAGSTPCNGVEGFNCYFPLQSYGFQPT
		aatcttgattctaaggttggtggtaattataattacctgtatagattgtttaggaagtctaatctcaaaccttttgagagagatatt<br>tcaactgaaatctatcaggccggtagcacaccttgtaatggtgttgaaggttttaattgttactttcctttacaatcatatggtttc<br>caacccacttag
G	420-540	DYNYKLPDDFTGCVIAWNSNNLDSKVGGNYNYLYRLFRKSNLKPFERDISTEIYQAGSTPCNGVEGF<br>NCYFPLQSYGFQPTNGVGYQPYRVVVLSFELLHAPATVCGPKKSTNLVKNKCVN
		gattataattataaattaccagatgattttacaggctgcgttatagcttggaattctaacaatcttgattctaaggttggtggtaat<br>tataattacctgtatagattgtttaggaagtctaatctcaaaccttttgagagagatatttcaactgaaatctatcaggccggtag<br>cacaccttgtaatggtgttgaaggttttaattgttactttcctttacaatcatatggtttccaacccactaatggtgttggttaccaa<br>ccatacagagtagtagtactttcttttgaacttctacatgcaccagcaactgtttgtggacctaaaaagtctactaatttggttaa<br>aaacaaatgtgtcaattag
H	481-540	NGVEGFNCYFPLQSYGFQPTNGVGYQPYRVVVLSFELLHAPATVCGPKKSTNLVKNKCVN
		aatggtgttgaaggttttaattgttactttcctttacaatcatatggtttccaacccactaatggtgttggttaccaaccatacaga<br>gtagtagtactttcttttgaacttctacatgcaccagcaactgtttgtggacctaaaaagtctactaatttggttaaaaacaaatg<br>tgtcaattag
I	475-500	AGSTPCNGVEGFNCYFPLQSYGFQPT

		gccggtagcacaccttgtaatggtgttgaaggttttaattgttactttcctttacaatcatatggtttccaacccacttag
**J**	520-540	APATVCGPKKSTNLVKNKCVN<br>gcaccagcaactgtttgtggacctaaaaagtctactaatttggttaaaaacaaatgtgtcaattag
**K**	660-680	YECDIPIGAGICASYQTQTNS<br>tatgagtgtgacatacccattggtgcaggtatatgcgctagttatcagactcagactaattcttag
**L**	660-710	YECDIPIGAGICASYQTQTNSPRRARSVASQSIIAYTMSLGAENSVAYSNN<br>tatgagtgtgacatacccattggtgcaggtatatgcgctagttatcagactcagactaattctcctcggcgggcacgtagtgtag ctagtcaatccatcattgcctacactatgtcacttggtgcagaaaattcagttgcttactctaataactag
**M**	990-1035	EVQIDRLITGRLQSLQTYVTQQLIRAAEIRASANLAATKMSECVLG<br>gaagtgcaaattgataggttgatcacaggcagacttcaaagtttgcagacatatgtgactcaacaattaattagagctgcaga aatcagagcttctgctaatcttgctgctactaaaatgtcagagtgtgtacttggatag
**N**	931-970	IGKIQDSLSSTASALGKLQDVVNQNAQALNTLVKQLSSNF<br>attggcaaaattcaagactcactttcttccacagcaagtgcacttggaaaacttcaagatgtggtcaaccaaaatgcacaagct ttaaacacgcttgttaaacaacttagctccaatttttag
**S21P2**	709-727	PSKPSKRSFIEDLLFNKV<br>CCCAGCAAGCCCAGCAAGAGAAGCTTCATCGAGGACCTGCTGTTCAACAAGGTGTGA
**S14P5**	552-570	TESNKKFLPFQQFGRDIA<br>ACCGAGAGCAACAAGAAGTTCCTGCCCTTCCAGCAGTTCGGCAGAGACATCGCCTGA

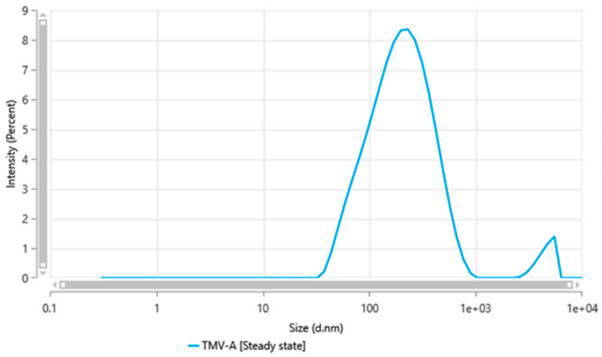

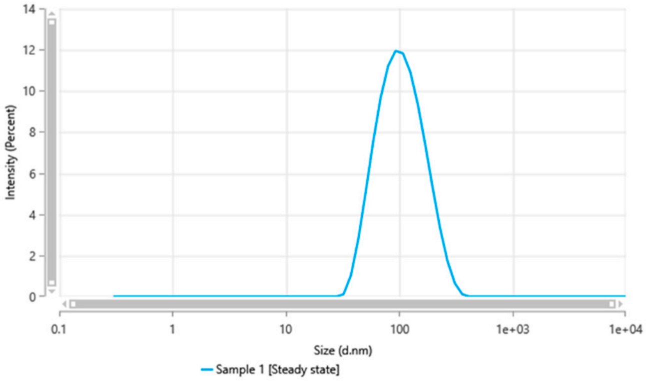

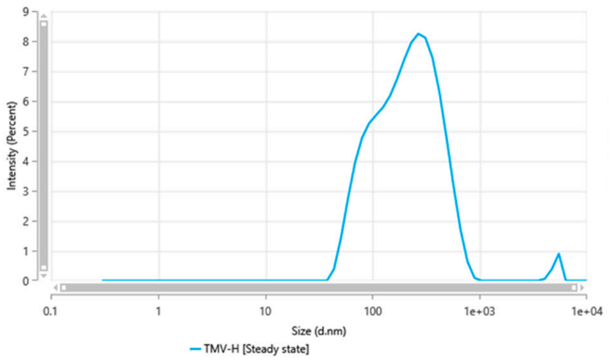
Intensity (Percent)
Size (d.nm)
TMV-H [Steady state]

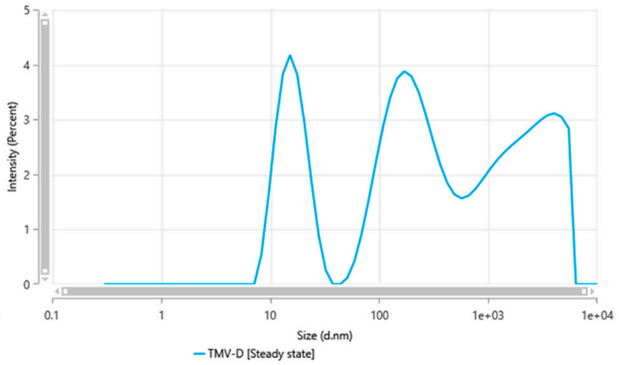
Intensity (Percent)
Size (d.nm)
TMV-D [Steady state]

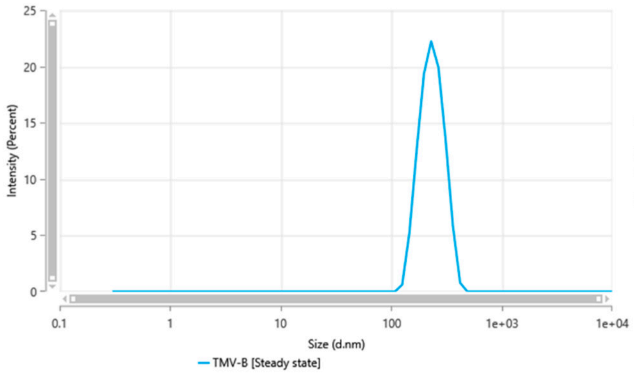
Intensity (Percent)
Size (d.nm)
TMV-B [Steady state]

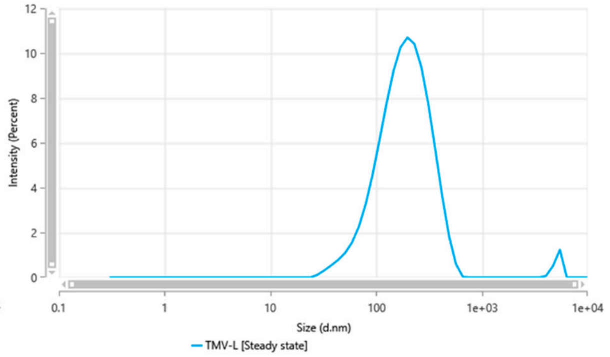
Intensity (Percent)
Size (d.nm)
TMV-L [Steady state]

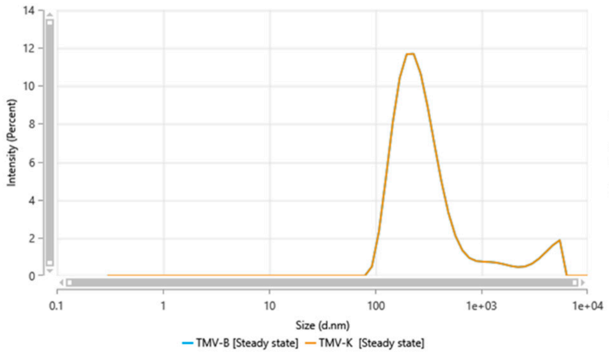
Intensity (Percent)
Size (d.nm)
TMV-B [Steady state]
TMV-K [Steady state]

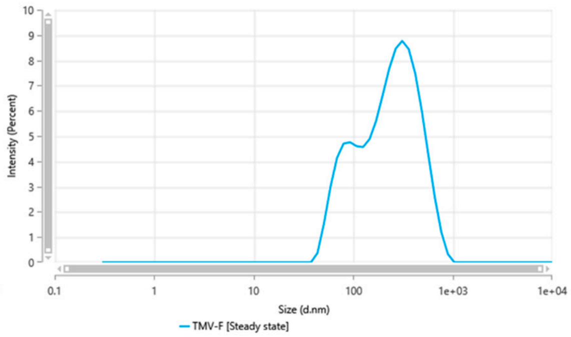
Intensity (Percent)
Size (d.nm)
TMV-F [Steady state]

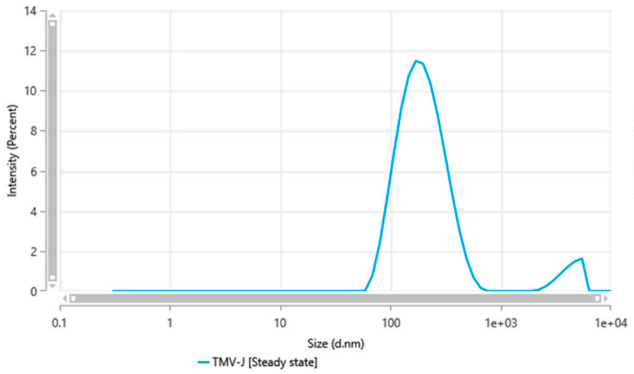
Intensity (Percent)
Size (d.nm)
TMV-J [Steady state]

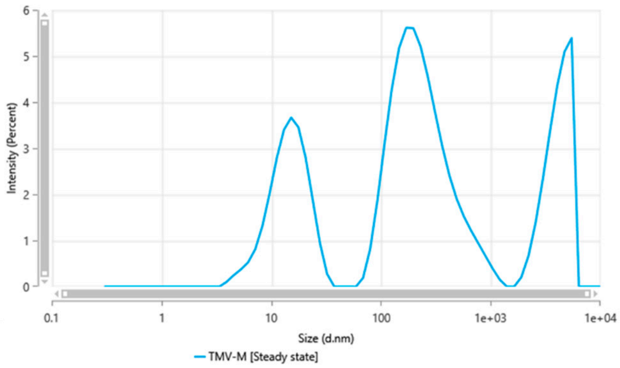
Intensity (Percent)
Size (d.nm)
TMV-M [Steady state]

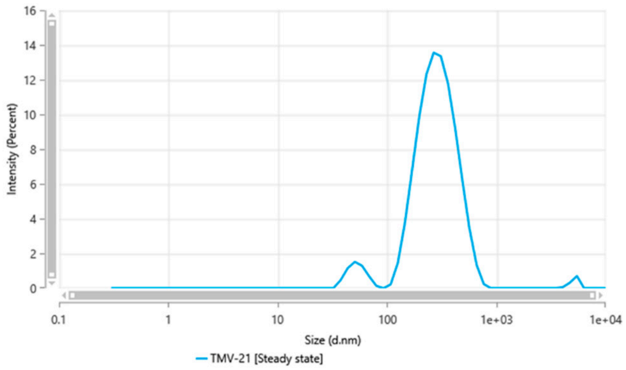
Intensity (Percent)
Size (d.nm)
TMV-21 [Steady state]

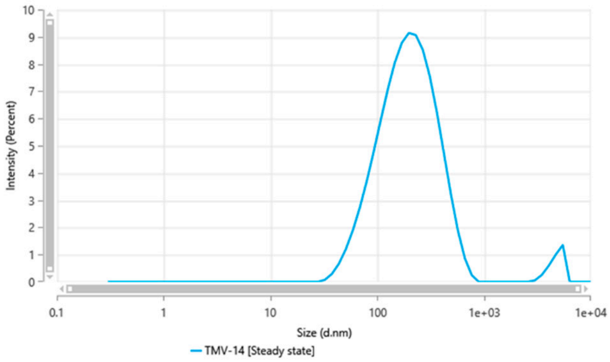
Intensity (Percent)
Size (d.nm)
TMV-14 [Steady state]

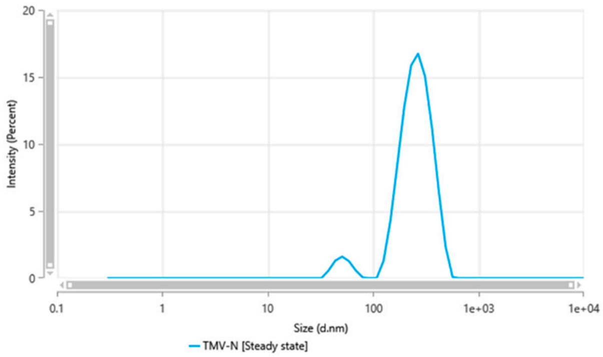
Intensity (Percent)
Size (d.nm)
TMV-N [Steady state]
